# Perspective: Emerging strategies for determining atomic-resolution structures of macromolecular complexes within cells

**DOI:** 10.1016/j.jsb.2021.107827

**Published:** 2021-12-14

**Authors:** Petar N. Petrov, Holger Müller, Robert M. Glaeser

**Affiliations:** a Department of Physics, University of California-Berkeley, Berkeley, CA 94720, USA; b Lawrence Berkeley National Laboratory, Berkeley, CA 94720, USA; c Department of Molecular and Cell Biology, University of California-Berkeley, Berkeley, CA 94720, USA

**Keywords:** Proteomics, Atomic-resolution readout, Tilt series, Focal series, Phase plate

## Abstract

In principle, electron cryo-tomography (cryo-ET) of thin portions of cells provides high-resolution images of the three-dimensional spatial arrangement of all members of the proteome. In practice, however, radiation damage creates a tension between recording images at many different tilt angles, but at correspondingly reduced exposure levels, *versus* limiting the number of tilt angles in order to improve the signal-to-noise ratio (SNR). Either way, it is challenging to read the available information out at the level of atomic structure. Here, we first review work that explores the optimal strategy for data collection, which currently seems to favor the use of a limited angular range for tilting the sample or even the use of a single image to record the high-resolution information. Looking then to the future, we point to the alternative of so-called “deconvolution microscopy”, which may be applied to tilt-series or optically-sectioned, focal series data. Recording data as a focal series has the advantage that little or no translational alignment of frames might be needed, and a three-dimensional reconstruction might require only 2/3 the number of images as does standard tomography. We also point to the unexploited potential of phase plates to increase the contrast, and thus to reduce the electron exposure levels while retaining the ability align and merge the data. In turn, using much lower exposures per image could have the advantage that high-resolution information is retained throughout the full data-set, whether recorded as a tilt series or a focal series of images.

## 1. Introduction

Electron tomograms of intact cells are nothing less than three-dimensional (3-D), *in situ* images of entire proteomes, as was perceptively recognized in (Baumeister, 2005). Nevertheless, for all but the largest structural elements within a cell, the ability to read this information out in an understandable form is currently limited by the still-incomplete development of electron cryo-microscopy (cryo-EM). Although electron microscopy has long been capable of producing atomic-resolution structures of materials that are robust against exposure to ionizing radiation, biological samples must be recorded with exposures that are too low to produce the signal-to-noise ratio (SNR) that is required to visualize structures at atomic resolution (Glaeser, 1971; Henderson, 1995).

Sub-tomogram averaging (STA) was introduced as a way to increase the SNR to the required level (Beck et al., 2007; Frangakis et al., 2002). Atomic-resolution structures were first achieved by STA for the capsid plus spacer peptide 1 region of Gag, using tomograms of purified, immature HIV-1 assemblies (Mattei et al., 2018; Schur et al., 2016). Similar technology has since been extended to produce atomic-resolution structures of ribosome particles within their native, cellular environment (Tegunov et al., 2021). The same capability should now be achievable for other structural elements of the cell, provided that they are large and recognizable enough. Extracting individual copies of the many thousands of smaller structural elements, however, remains challenging. Nevertheless, further development of cryo-EM technology is to be expected, offering hope that it will be possible to extend the high-resolution STA approach to more and more of the proteome.

Cellular high-resolution template matching (cHRTM) has recently been introduced as an alternative way to read out the positions and orientations of individual macromolecules (Rickgauer et al., 2017; Rickgauer et al., 2020). It uses already-known, atomic-resolution structures to generate templates, which are used to detect occurrences of the corresponding elements within cryo-EM images. cHRTM is appealing because it does not require averaging of data derived from multiple copies of a macromolecule, and because it provides a clear path to identifying small differences in individual composition or conformation. However, cHRTM is limited to finding elements for which one already has high-resolution structures, such as theoretical models or experimentally determined structures.

Whether using either STA or cHRTM, further improvements in the technology used for data collection will only help to reach the goal of imaging the full proteome. One such improvement may be to use a phase plate (Danev et al., 2014; Glaeser, 2013; Turnbaugh et al., 2021) rather than a large value of defocus to generate contrast at low spatial frequencies. A related goal of using a phase plate is to minimize oscillations in the contrast transfer function (CTF), to the extent that it is possible given the unavoidable variations in defocus that are associated with the thickness of the specimen itself. The benefit of doing so is to more fully recover the signal that is otherwise lost at spatial frequencies lying between the maxima in the power spectrum. A second area in which improvement is needed relates to the detective quantum efficiency (DQE) of the camera (McMullan et al., 2014). Values of the DQE should remain as close as possible to 1.0 out to atomic resolution, while retaining a large field of view, perhaps ~ 4 k × 4 k pixels or more. Finally, any remaining loss of signal, such as that due to beam-induced specimen motion, must be avoided at the highest values of resolution.

This PERSPECTIVE reviews a few of the technological issues (Beck and Baumeister, 2016) that currently make it difficult to achieve the goal of molecular readouts at atomic resolution in cryo-EM of intact cells, and it suggests some further advances that might now be considered. The focus is on improvements in both the instrumentation and the strategies used for data collection and analysis. At the same time, it is recognized that equally important issues remain in other areas, not covered here, such as sample preparation and software development.

### 1.1. Tilt-series tomography

As originally developed, electron tomography of cells assumed that cryo-EM images represented two-dimensional (2-D) projections of the object (Dierksen et al., 1993; Dierksen et al., 1992; Koster et al., 1992; Kremer et al., 1996; Ladinsky et al., 1999; O’Toole et al., 2002). Data are usually collected while tilting about a single axis, although the possibility of tilting about two orthogonal axes was also recognized from the beginning (Mastronarde, 1997). Images of biological specimens are extremely noisy, but high-resolution density maps can still be obtained by merging data from a large enough set of sub-tomograms, as mentioned earlier, provided that the particles have identical structures.

While single-axis tomography provides stunning images of samples (Mahamid et al., 2016; Medalia et al., 2002), provided that they are thinner than about 0.5 µm, multiple factors limit the resolution that can be achieved (without resorting to averaging) to values of 4 nm or so, which corresponds to sampling with a voxel size of 2 nm, estimated to be the smallest unit for which protein might be distinguished from water (Glaeser and Hall, 2011). Some of the limitations are practical issues that can, in principle, be overcome, such as the increase in beam-induced motion that becomes manifest as the sample is tilted. Other limitations, however, are more fundamental, such as the possible failure of the projection approximation when the resolution is increased; the increased fraction of inelastically scattered electrons, due to the increased thickness of material that the electron beam must go through as the sample is tilted; and the fact that radiation damage causes the structural information to decrease exponentially with electron exposure.

The approximation that cryo-EM images represent projections would seem to inherently limit the allowed thickness of a specimen to values that are less than the depth of field (DOF). For the values of resolution that are achievable without averaging, however, which – as mentioned above – are expected to be no more than about 4 nm, the DOF greatly exceeds the thickness limit due to inelastic scattering. Thus, without using STA, the useful sample-thickness values are limited by the loss of signal that is caused by inelastic scattering, rather than by the DOF.

As is explained in the Supplementary Material, on the other hand, the DOF decreases to about 250 nm at a resolution of 1 nm, and it drops to only about 25 nm as the resolution approaches 0.3 nm. When STA is used with the intent to obtain density maps at atomic resolution, one might thus expect the projection approximation to fail for most sample thicknesses of interest in tomography of cells. Fortunately, STA also opens the door for applying separate CTF corrections to each sub-tomogram (Galaz-Montoya and Ludtke, 2017; Galaz-Montoya et al., 2015). Nevertheless, the question remains whether applying some form of Ewald sphere reconstruction (or wavefront reconstruction) to images in the tilt-series, and not just to individual sub-tomograms, would improve the resulting 3-D maps, making it easier to visualize structures at atomic resolution. More is said about this question in Section 1.4 on Deconvolution Microscopy, below.

The possibility of multiple elastic scattering occurring for thicker specimens, which also is not accounted for by the projection approximation, is a second issue that might cause concern. Since the thickness of specimens is generally kept to values less than the mean-free-path for inelastic scattering, however, and since the cross section for inelastic scattering is about three times greater than that for elastic scattering, there is a good argument that multiple elastic scattering will generally not be a problem for tomography of biological specimens. It nevertheless is true that structure factors for large objects, on the scale of ribosomes, are very strong at low resolution. As a result, the image contrast of low-resolution features is expected to deviate significantly from being linear in the total projected mass when a 90-degree phase plate is used – see Figure 7a in (Danev et al., 2009). A simple work-around, should a problem arise, might be to filter out the lowest frequencies when computing an atomic-resolution map, as is usually done in X-ray crystallography.

### 1.2. Optimizing the strategy for tomographic data collection

The fact that the high-resolution information decays more rapidly with electron exposure than does low-resolution information (Baker and Rubinstein, 2010) means that only the first several images in a tilt series will contribute high-resolution features. As a result, the high-resolution portions of data set will be incomplete – i.e. not present for most of the tilt series – as well as being noisy. Is there, then, an optimal way to record a tomographic series of images, in order to best recover a complete, high-resolution data set?

Using a dose-symmetric scheme (Hagen et al., 2017) has become standard practice in cryo-tomography (cryo-ET): high-resolution information is confined to a limited set of central sections in Fourier space, which are symmetrically distributed on either side of the section corresponding to the image of an untilted specimen. Because they are recorded with very little specimen tilt, these data are affected to the least extent by beam-induced motion of the specimen.

The use of data sets collected with a smaller range of tilt angles, for example ± 45° or even as low as ± 30°, has also been investigated, but this strategy is not yet routine. In an early attempt (Schur et al., 2015), a resolution of ~8 Å was achieved for Rous-sarcoma virus Gag polyprotein, assembled into large, virus-like particles. While this work represented a major step forward, it did not yet use a direct-detection camera, and much further improvement could now be expected. Indeed, a resolution of ~3.96 Å has since been achieved for purified single particles, consisting of a ~300 kDa homohexameric complex of a dNTPase, using a tilt series confined to only ±36° (Bouvette et al., 2021).

Inspired by earlier attempts to do single-particle tomography (Bartesaghi et al., 2012; Galaz-Montoya et al., 2015), the question has even been raised whether it might be more effective to first record images of untilted specimens with relatively high electron exposures, for example 20 e/Å^2^ or perhaps even more, followed by recording the usual, dose-symmetric tilt series (Sanchez et al., 2020; Song et al., 2020). The goal in doing so is to obtain data corresponding to one central section in Fourier space with as high a SNR as possible, while still recovering a tomogram at the usual resolution, i.e. approaching ~ 4 nm. Such an approach effectively aims to combine the best of single-particle cryo-EM and cryo-ET. Although it contributes little high-resolution information, such a tomogram adds invaluable information about the positions of particles, including their relative z-heights, as well as providing constraints on the relative orientations of the macromolecular complexes.

The use of relatively thick, untilted specimens carries with it the fact that structural information contributed by elements above and below the object of interest represents unwanted background in the images of untilted specimens. We point out that, in principle, the low-resolution parts of this background can be subtracted from the initial images of untilted specimens once the desired sub-volumes are identified.

**Fig. 1.**
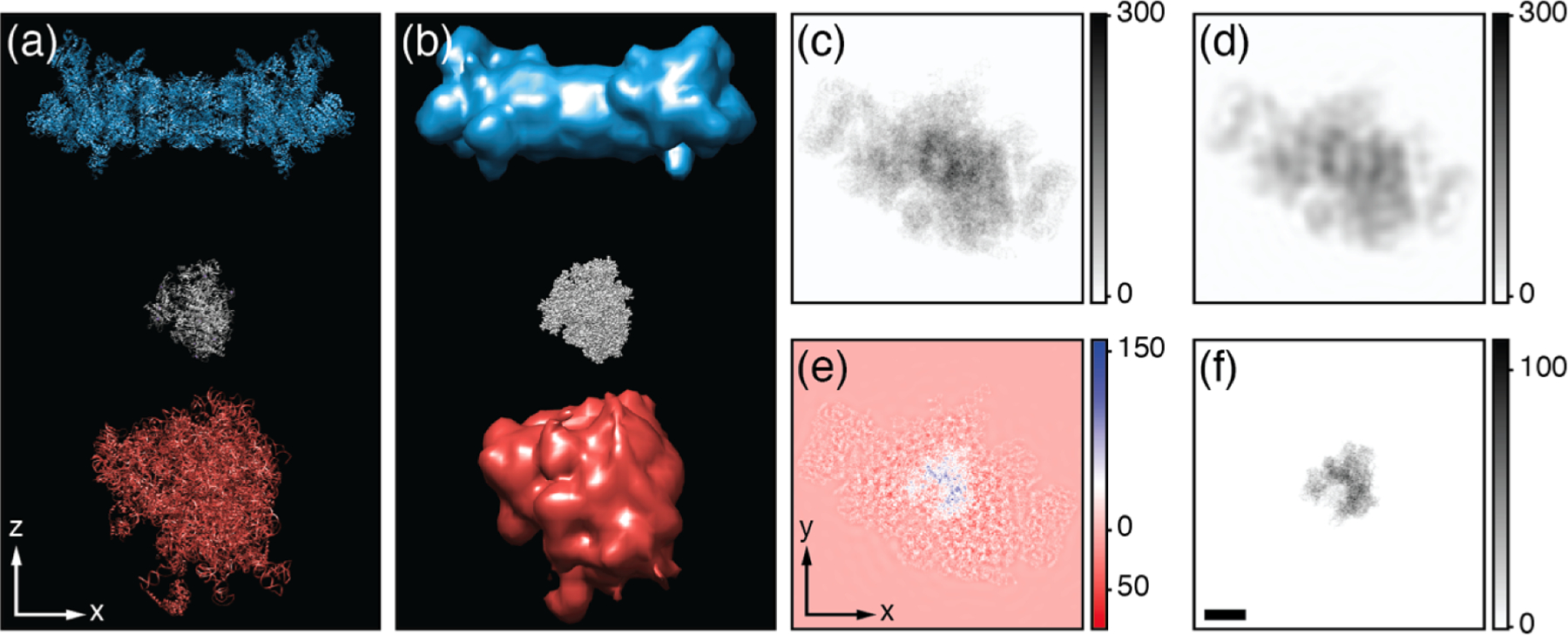
Schematic illustration of proposed low-resolution background-subtraction strategy. (a) X-Z view of a totally artificial phantom consisting of a 26S proteasome (PDB 5gjr), an RNA polymerase II (PDB 1i3q), and a eukaryotic ribosome (PDB 4v6x). The optical axis of the microscope is along Z. (b) Density map corresponding to (a) with the proteasome and ribosome shown at 40 Å resolution and the polymerase shown at 2 Å resolution. (c) X-Y projection of all three proteins at 2 Å resolution. (d) X-Y projection of the proteasome and ribosome at 40 Å resolution. (e) Difference between (c) and (d), which reveals the polymerase atop a high-frequency signature that necessarily remains after background subtraction. Note that a color scale, rather than a simple grey scale, is used for panel (e) in order to display both positive and negative values that are generated in the difference between intensities. (f) The X-Y projection of a lone polymerase at 2 Å resolution, for comparison with (e). Scale bar in (f) is 10 nm, and (c)-(f) are at the same scale. Color bar units are arbitrary. The effects of solvent, CTF, missing wedge, and noise were neglected here, as the goal is to illustrate just the concept of background subtraction.

To do this subtraction, properly weighted data from the image of the untilted specimen would have to be included with data from the tilt series when reconstructing a tomogram. As is indicated schematically in Fig. 1, the region of interest would then be masked out from the resulting tomogram, and the projection of the remaining material would be subtracted from the initial images of untilted specimens. Building on the idea of masked refinement (Bai et al., 2015; Ilca et al., 2015; Zhou et al., 2015), the possibility then exists to merge these background-subtracted projection data in the same way as is done for single-particle analysis (SPA).

Unfortunately, high-resolution structural information contributed by elements above and below objects of interest also represents unwanted background in the image of the untilted specimen. Furthermore, this high-resolution background cannot be subtracted, since it no longer exists by the time that the tomographic series of images is recorded. High-resolution background, contributed by elements above and below extracted sub-volumes, is also present in tomographic data sets recorded in the usual way, of course, with an important exception: these high-resolution features are distributed over several central sections, rather than in just a single central section.

At present the question remains unanswered whether it is better to record a single, high-resolution image of an untilted specimen or to distribute the initial electron exposure over at least a limited number of high-resolution images. If the latter is true, the good news is that a phase plate is expected to reduce the exposure needed to align images obtained at successive tilt angles, and thus the “safe” electron exposure of about 20 e/Å^2^ can be distributed over a larger range of tilt angles.

It is anticipated that the use of a phase plate might improve the quality of tomographic data sets in general, as well as that of the hybrid, “single-particle”/tomographic data sets envisioned above. Regrettably, data obtained with the Volta phase plate (VPP), initially used to demonstrate the stunningly improved tomograms that are possible (Mahamid et al., 2016), has often proved to be difficult to process. In addition, data obtained with the VPP seems to suffer from an unexplained loss of signal at high resolution (Buijsse et al., 2020). It thus is hoped that the recent development of a “laser phase plate” (LPP) (Schwartz et al., 2019; Turnbaugh et al., 2021) will be free of the shortcomings encountered with the VPP, and that it will allow one to more fully realize the goal of recovering all of the information carried by the elastically scattered electrons.

### 1.3. High-resolution template matching

High-resolution template matching has been proposed as an alternative to STA as a way to read out the content of cells at atomic resolution (Lucas et al., 2021; Rickgauer et al., 2017; Rickgauer et al., 2020). In this approach, the template is a noise-free, 2-D image of a given macromolecule that is computed for a physically realistic model of the experimental imaging conditions. For a given particle, many such 2-D templates (“projections”) are then used to exhaustively search for matches that might be found within 2-D, experimental images.

Current implementations of this approach use electron exposures of 20 e/Å^2^ or more to record just a single image of an untilted specimen. As mentioned previously, use of untilted specimens minimizes the loss of information caused by beam-induced specimen motion, which helps to recover the maximum SNR at high resolution. Including the defocus value as an adjustable parameter not only maximizes the template correlation when correct matches are found, but it also serves to recover information about the 3-D positions of target molecules.

These single, 2-D images are still very noisy, however, and even large particles can seldom be recognized by eye because their surroundings are very crowded. Nevertheless, it has been shown (Rickgauer et al., 2017) that it is possible to use such images to read out atomically precise locations and orientations of searched-for particles. A “true” match is indicated by a peak in the cross-correlograms with a threshold SNR of 8 or higher, sufficient to suppress false positives (Rickgauer et al., 2017).

Since only a “forward transform” is used to convert the 3-D, atomic-resolution structure of a particle into a 2-D template, this approach is not subject to the depth-of-field (DOF) limitation that might occur when doing the inverse transform from many 2-D images recorded with different views. Other limitations on the sample thickness, however, such as loss of signal due to inelastic scattering, or the accumulation of excessive phase modulation, will still remain.

A major advantage of the template-matching approach is that it does not require that data be merged from many thousands of identical particles. Instead, it is possible to read out the locations and orientations of individual particles, even those that are present in cells at very low copy number. What is nevertheless required is that the particle size be large enough, as is further addressed below, and that sufficiently large portions of the structures of the template and the target match at atomic resolution. On the other hand, the range of templates used can include conformational states computed by molecular dynamics or by fold-prediction methods (Baek et al., 2021; Jumper et al., 2021), and thus one is not limited to using templates that are already included in experimental data bases.

While an exhaustive search is guaranteed to find the optimal fit of a given template to the experimental data, that approach is also computationally intensive; indeed, for many it will be prohibitively so. Ten million or more templates may be needed to search one image of a thick sample for just one type of structure, without orientation restrictions, causing the number of cross-correlation values to approach 10^15^ (Rickgauer et al., 2020). Significant progress has been made to address this issue, however, by using Graphics Processing Units (GPUs) to accelerate the throughput (Lucas et al., 2021). Another alternative might be to use the high-performance computing infrastructure as a server, supported by dedicated funding as are synchrotron beamlines or cryo-EM National Service Centers.

High-resolution template matching has already been used to successfully read out the locations and orientations of the 350 kDa head region of ribosomal small subunits in thin regions at the margins of mouse embryonic fibroblast cells, cultured on EM grids (Rickgauer et al., 2020). Going even further, it has been shown that compositional differences as small as 24 kDa can be read out when the search is restricted to the possible location and orientation that is expected from prior information. Even further improvement can be expected when a phase plate is used to record images; when the DQE of cameras is improved significantly while retaining a large field of view; when recovering high-resolution information (currently lost due to beam-induced motion) more effectively; and when using a cold FEG (Nakane et al., 2020) or a gun monochromator (Yip et al., 2020) to improve the temporal coherence at very high resolution.

### 1.4. Deconvolution and focal series

Deconvolution techniques, in which an improved estimate of a 3-D object is obtained by deconvolving the point spread function (PSF) of the microscope from the raw image data, have been widely adopted in fluorescence microscopy. The goal of such techniques is to leverage knowledge about the optical system to computationally undo the blurring of the object by the microscope and thus enhance image contrast. Algorithms adopted from fluorescence microscopy have already been applied to (incoherent) scanning transmission electron microscopy (STEM) (Ramachandra and de Jonge, 2012) and, more recently, introduced to cryogenic STEM tomography (Waugh et al., 2020).

Volumetric deconvolution can be performed using either data obtained by tilting the specimen or, leveraging the shallow DOF of optical microscopes, data obtained by simply scanning the focal plane. While focal series do not, in general, contain enough information for a 3D reconstruction (Streibl, 1985), enough information can be obtained by using the extended-depth of field approach, a 2-D reconstruction method that shows all specimen features in focus (Forster et al., 2004; Hovden et al., 2011). Furthermore, it may even be possible that a focal series alone does allow for a 3D-reconstruction for single-scattering objects (Streibl, 1985).

Use of deconvolution techniques for coherent imaging schemes such as brightfield optical microscopy has proven more challenging than for incoherent imaging schemes, however, because it requires characterization of both the phase and amplitude PSFs (Hernández Candia and Gutiérrez-Medina, 2014; Jenkins and Gaylord, 2015). However, cryo-EM operates in the weak-phase regime, for which image formation is well-approximated as being linear in the phase of the wave function transmitted through the object. In light of this simplification, it may be reasonable to further investigate the adoption of tools that have proven to be so successful in the context of fluorescence microscopy. An entropy-regularized approach to 3-D deconvolution, like that used by (Croxford et al., 2021) in an attempt to fill in the missing wedge associated with tilt-series electron tomography, seems a promising one to also try with focal series data. While a focal series has no “missing wedge problem” as such, it is expected that regularization will help to make its inverse transform more robust in the presence of low SNR.

**Fig. 2.**
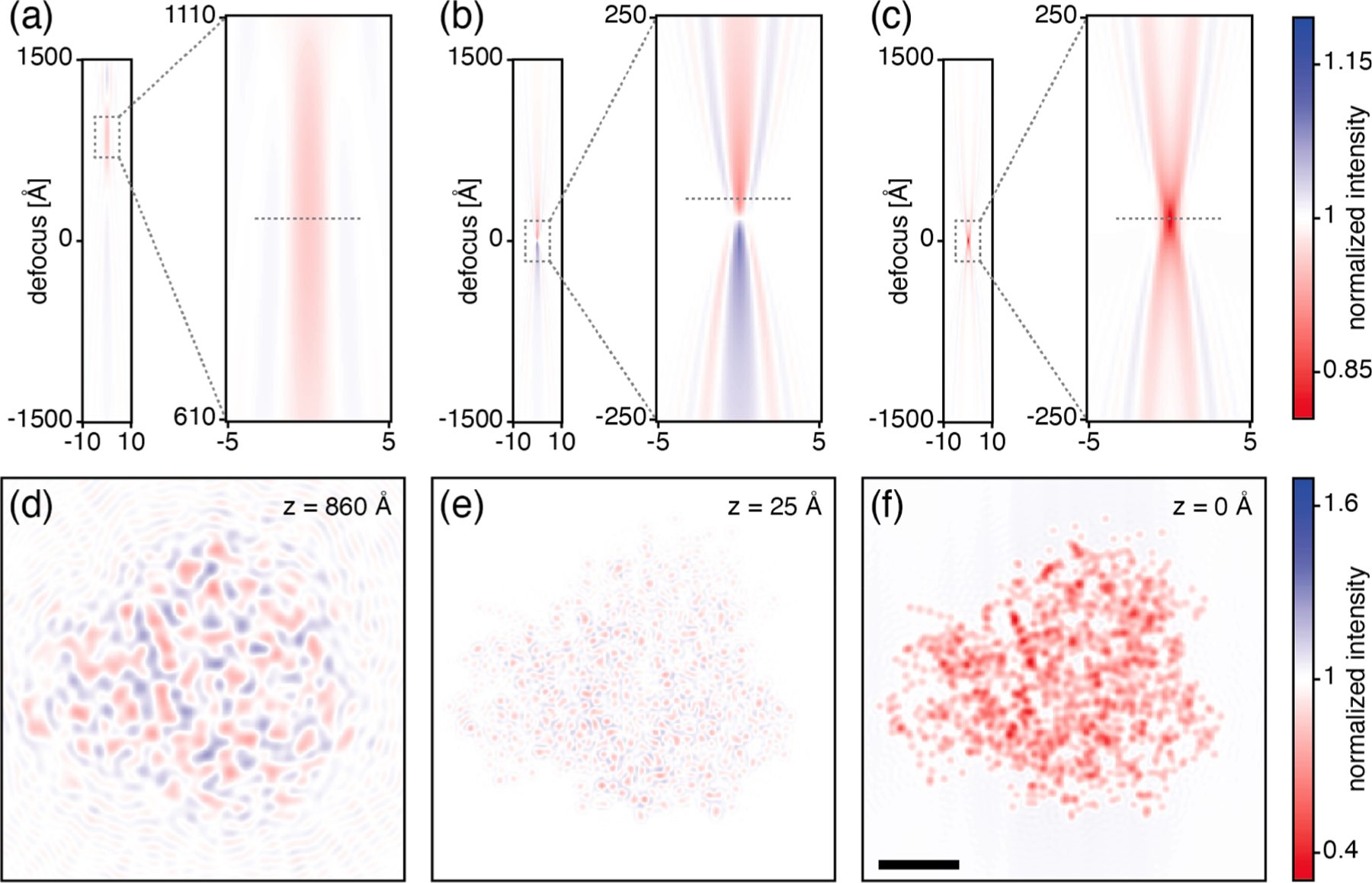
Demonstration of potential improvements provided by aberration-corrected phase contrast TEM. (Top row) The image of a single carbon atom is simulated with different amounts of defocus, and line scans through the corresponding X-Y views (images) are stacked in the vertical direction. The resulting image stack is shown for a microscope without aberration correction or phase plate (a), with aberration correction only (b), and with both aberration correction and a laser phase plate (c). Insets of the areas shown with dashed gray rectangles show the high-contrast region near the Scherzer defocus for each configuration. Axis labels are in units of ångstroms. (Bottom row) Panels (d-f) show simulated, X-Y images of a myoglobin molecule. The myoglobin molecule is simulated with its center of mass being at the highest-contrast z plane in the panel above it (value indicated in the top right of each panel, indicated with a dashed gray line in (a)-(c)). In this simulation, the protein is not solvated (i.e. it is in vacuum), the intent being only to illustrate the potential benefit of combining a phase plate with an aberration corrector. Scale bar is 10 Å and applies to (d)-(f). Images omit noise and are normalized so that the background intensity is 1. Microscope parameters are listed in Table 1.

The advent of aberration correctors for electron microscopes leads, in fact, to an opportunity for optical sectioning (Borisevich et al., 2006), which in turn could be leveraged when taking a deconvolution approach (Tang et al., 2006). Compared with that of a conventional cryo-EM (Fig. 2a), the DOF can be greatly reduced, even to less than 10 nm (Fig. 2b, Supplementary Material). The incorporation of recently-developed phase contrast techniques (Danev et al., 2014; Turnbaugh et al., 2021) into an aberration-corrected TEM would further improve the shape and limit the extent of the 3-D PSF (Fig. 2c).

For an in-focus object, the resulting contrast transfer function (CTF) might approach the ideal of having a value near to 1.0 over a large band of spatial frequencies, extending out to very high resolution, a situation that would provide the best possible SNR for deconvolution. Additionally, the possibility to quickly toggle a phase plate on and off may also facilitate the measurement of both the phase and amplitude PSFs, providing complete PSF information for the deconvolution of scattering samples that go even beyond the weak-phase or single-scattering limits.

Sample thickness values often exceed the DOF in fluorescence microscopy experiments, where focal series can be used in place of tilt series in order to gain information in 3-D (Agard, 1984). As in fluorescence microscopy, it can be imagined that optical sectioning of cryo-EM specimens would enable significant background reduction when the sample thickness exceeds the DOF. Reduction of the background above and below the DOF might even enable one to look inside large macromolecules. The curvature of the Ewald sphere would then play a significant role in image formation, however, and that needs to be dealt with explicitly (Chen et al., 2021; DeRosier, 2000). It seems that a natural way to do so is to generalize the simple solution for the Ewald sphere problem, presented in Section 2.6 of (DeRosier, 2000), to include multiple values of defocus.

However, the radiation damage accrued while imaging a thick object in a focal series requires that the exposure per image must be reduced to approximately 20/*N* e/Å^2^, where *N* is the number of images in the data set. While the value of *N* is currently limited to about 20 in tilt-series based cryo-ET, this might be increased significantly by the use of a phase plate, because the low-resolution signal is then very much greater, making the images easier to align. In addition, images in a focal series may be much easier to align at lower exposure levels than those in a tilt series, and thus the value of *N* may be increased even further.

Finally, it is important to point out that Shannon sampling requires only 2D/d images in order to reconstruct an object of size D at a resolution of d, which is only 2/π the number needed to satisfy the Crowther criterion for uniaxial tomography (Crowther et al., 1970). We therefore consider it worthwhile to investigate whether focal series reconstruction can approach the Shannon sampling limit, in which images are recorded from 2D/d physical sections of an object.

Nevertheless, a hybrid imaging scheme involving both tilts and focal series may be more beneficial than using just a focal series of images, as was illustrated recently for aberration-corrected STEM (Yalisove et al., 2021) and explored theoretically in TEM (Gureyev et al., 2021; Ren et al., 2020). Such a hybrid approach could still benefit from reducing the number of tilts, and, by adding a known amount of parallax, it could better exploit the range of z positions and orientations of protein copies in a cryo-tomography sample.

## Supplementary Material

Supplementary material

## Figures and Tables

**Table 1:** Parameters used in Fig. 2. Comparison of parameters for uncorrected and Cs-corrected microscopes; in both cases it is assumed that a gun monochromator is used, so that the effect of the temporal coherence envelope will be the same. Laser phase plate parameters (last two rows) are added to the Cs-corrected microscope column for consistency with Fig. 2, but the laser phase plate can be used without a Cs corrector.

	Uncorrected	Cs-corrected
**HT**	300 kV	300 kV
**Cs**	2.7 mm	0
**Cc**	2.7 mm	8 mm
**focal length**	3.5 mm	19.8 mm
**energy spread (FWHM)**	0.1 eV	0.1 eV
**beam divergence (FWHM)**	2.5 μrad	2.5 μrad
**cavity numerical aperture**	–	0.04
**peak phase shift**	–	90 deg
