# Supplementary material for: Perspective: Emerging strategies for determining atomic-resolution structures of macromolecular complexes within cells
Source: J Struct Biol. Author manuscript; Available in PMC 2022 Apr 4. (PMC8978977; doi:10.1016/j.jsb.2021.107827)
Supplement: Supplementary material [file NIHMS1783260-supplement-Supplementary_material.docx]

SUPPLEMENTARY MATERIAL

**PERSPECTIVE: EMERGING STRATEGIES FOR**

**DETERMINING ATOMIC-RESOLUTIION STRUCTURES**

**OF MACROMOLECULAR COMPLEXES WITHIN CELLS**

Petar N. Petrov, Department of Physics, University of California-Berkeley, Berkeley, CA 94720, USA.

Holger Mueller, Department of Physics, University of California-Berkeley, Berkeley, CA 94720, USA, and Lawrence Berkeley National Laboratory, Berkeley, CA 94720, USA.

USA.

Robert M. Glaeser, Department of Molecular and Cell Biology, University of California-Berkeley, Berkeley, CA 94720, USA .and Lawrence Berkeley National Laboratory, Berkeley, CA 94720, USA.

The depth of field (DOF) is defined here to be the *difference in defocus values*, for two points that are separated from one another along the optical axis, for which the phase aberration, $\gamma(s)$, which itself is given by

$\gamma\left( s \right)=2\pi\left[ \frac{C_{s}\lambda^{3}\frac{1}{d^{4}}}{4}-\frac{\Delta z\lambda}{2}\frac{1}{d^{2}} \right]$,

differs by $\frac{\pi}{2}$. In this equation, $C_{s}$ is the coefficient of spherical aberration, *d* is the resolution, λ is the electron wavelength, and $\Delta z$ is the amount by which the objective lens is defocused.

The phase shift due to defocus depends upon both the electron wavelength and the square of the resolution, of course, and one can easily show that the DOF, as defined here, is given by the equation

$DOF=\frac{d^{2}}{2\lambda}$ ,

where, as before, *d* is the resolution and λ is the electron wavelength. In the case of 300 keV electrons, λ is ~0.02 Å, and thus the DOF is ~4 µm at a resolution of 4 nm. As mentioned in the text, however, the DOF drops to a value of only about 250 nm at a resolution of 1 nm, and to about only 25 nm as the resolution approaches 0.3 nm
